# Predictive role of tear metabolomics in delirium during anesthesia emergence and postoperative period in elderly patients after abdominal surgery

**DOI:** 10.3389/fmolb.2026.1705024

**Published:** 2026-06-04

**Authors:** Jing Wang, Mingju Xiang, Pingzhou Cheng, Yingsong Zhu, Tingting Fan, Bin Shu, Jie Chen, Guangyou Duan, Jian Li, He Huang

**Affiliations:** 1 Department of Anesthesiology, The Second Affiliated Hospital of Chongqing Medical University, Chongqing, China; 2 Department of Anesthesiology, People’s Hospital of Chongqing Hechuan, Chongqing, China

**Keywords:** biomarkers, emergence delirium, metabolomics, postoperative delirium, tear fluid

## Abstract

**Objective:**

To investigate characteristic tear metabolite changes in patients with postoperative delirium using tear metabolomic analysis and evaluate their potential as biomarkers for diagnosing delirium.

**Methods:**

In this study, 141 elderly patients undergoing abdominal surgery were enrolled. Tear samples were collected after anesthesia induction and before completion of surgical suturing. Delirium was assessed as emergence delirium (ED) in the PACU and ward delirium (WD) during the postoperative ward stay. Tear metabolites were analyzed by ultra-high-performance liquid chromatography-Orbitrap exploris-mass spectrometry.

**Results:**

Based on the occurrence of ED and WD, patients were stratified into two matched pairs of subgroups: the ED group (n = 32) and its matched non-ED control group (n = 32), while the WD group (n = 34) and its matched non-WD control group (n = 34), respectively. Metabolomic analysis showed that five and three important metabolic pathways were respectively associated with ED and WD. A decrease in arachidonic acid levels before surgery and an increase in dopamine levels after surgery may predict ED occurrence, whereas a decrease in glyoxylic acid levels after surgery may predict WD. The receiver operating characteristic curve analysis yielded an area under the curve values of 0.88 (95%CI: 0.78–0.97) and 0.88 (95%CI: 0.80–0.97) for preoperative and postoperative differential metabolite ED models, respectively. The WD model predictive efficiency reached 0.75 (95%CI: 0.63–0.87) and 0.78 (95%CI: 0.67–0.89), respectively.

**Conclusion:**

In this study, changes in metabolic pathways and metabolite levels in tears associated with postoperative delirium were identified. The delirium prediction models established based on tear metabolomics show good predictive efficacy.

## 1 Introduction

Postoperative delirium is a prevalent postoperative complication among elderly patients during the recovery phase from general anesthesia and within the postoperative ward. Its primary manifestations are acute onset, fluctuating symptoms, impaired attention, confusion, and an altered state of consciousness (Oh et al., 2017). Delirium after surgery during emergence (ED), a subtype of postoperative delirium, occurs during the recovery from general anesthesia, such as within the post-anesthesia care unit. ED presents with acute and fluctuating changes in consciousness and attention, with studies reporting incidence rates ranging from 37% to 45% (Zhang et al., 2020; Neufeld et al., 2013; Sharma et al., 2005). Research demonstrates that postoperative delirium is associated with higher complication rates and may foreshadow delirium episodes in subsequent hospitalization. Moreover, ED is associated with cognitive function decline following discharge (Neufeld et al., 2013). Delirium after surgery in ward (WD), mostly occurs 1–7 days after surgery, with an incidence ranging from 30.9%–36% (Sharma et al., 2005; Choi et al., 2017; Miyagawa et al., 2017; Kim et al., 2022). WD can increase postoperative complications incidence, prolonged hospital stays, increased medical costs, and postoperative mortality rates (Marcantonio, 2012; Liu and Wang, 2015).

Early identification and diagnosis of patients at high risk for delirium are essential for its prevention and treatment. Owing to the precise pathophysiological mechanisms underlying delirium remaining unknown, its diagnosis is currently reliant on medical history and consultation, primarily using various delirium assessment tools. The Diagnostic and Statistical Manual of Mental Disorders (Fifth Edition; DSM-5) provides the diagnostic framework for these assessment instruments, among which the 3-Minute Diagnostic Interview for Confusion Assessment Method (3D-CAM) and Confusion Assessment Method for the Intensive Care Unit (CAM-ICU) are widely used implementations (Wilson et al., 2020; Mattison, 2020; Tang et al., 2023). However, clinical biomarkers for the diagnosis and prediction of postoperative delirium remain lacking. Studies show that brain structure and function have been monitored and evaluated by analyzing blood and cerebrospinal fluid (CSF) or by applying other imaging techniques such as electroencephalography (EEG), computed tomography (CT), and magnetic resonance imaging (MRI) (Cunningham et al., 2019; Liang et al., 2023; White et al., 2021; Akhtar et al., 2023). Although these methods offer valuable insights, they also present significant drawbacks. For example, collecting blood and CSF is invasive and subject to various influencing factors. EEG signals are prone to interference, and CT and MRI require patient movement, which can be challenging. Therefore, noninvasive and easy-to-obtain methods are urgently required to detect biomarkers of WD.

As a body fluid, tears are an ideal biomarker source owing to their convenient collection and noninvasive nature. Tears have attracted increasing attention for disease biomarker research and they are expected to become the next routine biochemical diagnostics (Barmada and Shippy, 2020). First, the lacrimal glands located within the eyelids produce tears mainly via filtration of blood plasma components. As plasma circulates throughout the tissues and organs of the body, tears can also carry biomarkers that reflect systemic diseases. Second, as plasma is filtered and secreted by the lacrimal gland, certain systemic disease biomarkers in plasma may become concentrated in tears. For example, Wang et al. used electrochemical sensors to analyze β-Amyloid (Aβ), a key biomarker for Alzheimer disease (AD), in blood and tear samples from healthy individuals. They found that Aβ concentration in tears was approximately 10 times higher than that in blood (Wang et al., 2021). Additionally, studies show that AD, Parkinson’s disease, multiple sclerosis, and other central nervous system disorders have been studied using tears (Gharbiya et al., 2023; Lin et al., 2022; Cicalini et al., 2019). Hence, we hypothesize that tears could serve as a predictive specimen for postoperative delirium in elderly patients. Therefore, this study aims to use ultra-high performance liquid chromatography (UHPLC)-Orbitrap Exploris-Mass spectrometry to conduct nontargeted metabolomic analysis of tear samples from geriatric patients receiving general anesthesia for abdominal procedures. This study could identify biomarkers associated with postoperative delirium, offering more insights into its pathophysiological mechanism and generating new ideas for its diagnosis and prevention.

## 2 Methods

### 2.1 Patients and setting

This study was designed as an observational trial. The research protocol received ethical clearance from the Ethics Committee at the Second Affiliated Hospital of Chongqing Medical University (Approval ID: 2022–122), with ethics registration completed on clinicaltrials.gov (NCT05548153) before the initiation of the study. The study participants were recruited from patients undergoing surgical procedures at the Second Affiliated Hospital of Chongqing Medical University from August 2022 to January 2023. Eligible subjects met the following criteria: age ≥65 years, planned elective abdominal surgery requiring general anesthesia, and American Society of Anesthesiologists (ASA) physical status classification I-III. Exclusion criteria were severe vision, auditory, or language impairment, or any other condition that impaired communication; history of preoperative mental illness or cognitive impairment; a preoperative Mini-Mental State Examination (MMSE) score <24; and long-term use of sedative drugs, history of drug abuse or a history of alcoholism. All participants provided written informed consent prior to study enrollment. The study protocol and all de-identified data are available upon reasonable request from the corresponding author via email.

### 2.2 Anesthesia management

All patients fasted for 8 h and were restricted from drinking water for 2 h before surgery. Upon entering the operating room, intravenous access was established and comprehensive monitoring was initiated, including continuous electrocardiogram (ECG), heart rate, blood pressure measurement, and peripheral oxygen saturation (SpO_2_) monitoring. Anesthetic depth was assessed using the bispectral index (BIS) system. Rapid sequence induction was conducted using midazolam (0.02–0.04 mg/kg), sufentanil (0.3–0.6 μg/kg), propofol (1.5–2.5 mg/kg), and rocuronium bromide (0.6 mg/kg). Following intubation, the anesthesia machine was connected to mechanical ventilation and respiratory parameters were set to: 6–8 mL/kg tidal volume, 10–14/min respiratory rate, 1:2 inspiratory-to-expiratory ratio, and partial pressure of end-tidal carbon dioxide (PETCO_2_) maintained at 30–40 mmHg.

Anesthesia maintenance was achieved through combined sevoflurane inhalation (1.0%–1.3%) and intravenous administration of propofol (1–4 mg/kg/h) plus remifentanil (0.1–0.2 μg/kg/min), with dosages titrated to maintain BIS values between 45–60. Intermittent intravenous rocuronium bolus was administered to maintain appropriate muscle relaxation during surgery. Heart rate and blood pressure were maintained within ±20% of the baseline levels, with vasoactive drugs administered when necessary.

Patients selected their analgesic modality based on their individual preoperative pain sensitivity and tolerance levels. All patients opted for transversus abdominis plane (TAP) block, with a subset additionally receiving supplementary patient-controlled intravenous analgesia (PCIA). Postoperatively, all patients uniformly received ultrasound-guided bilateral TAP blocks, with 20 mL of 0.375% ropivacaine injected per side. For those who voluntarily elected to receive supplemental PCIA, a solution containing 2 μg/kg sufentanil and 2 mg/kg flurbiprofen axetil in normal saline (total volume 100 mL) was provided, with the following parameters: 2 mL loading dose, 2 mL/h continuous infusion, 2 mL bolus doses (10-min lockout interval), and a maximum hourly limit of 0.1–0.2 mL/kg. For patients who declined PCIA, postoperative pain management measures were determined by ward physicians.

### 2.3 Assessment and standardized intervention strategies for postoperative delirium

**FIGURE 1:**
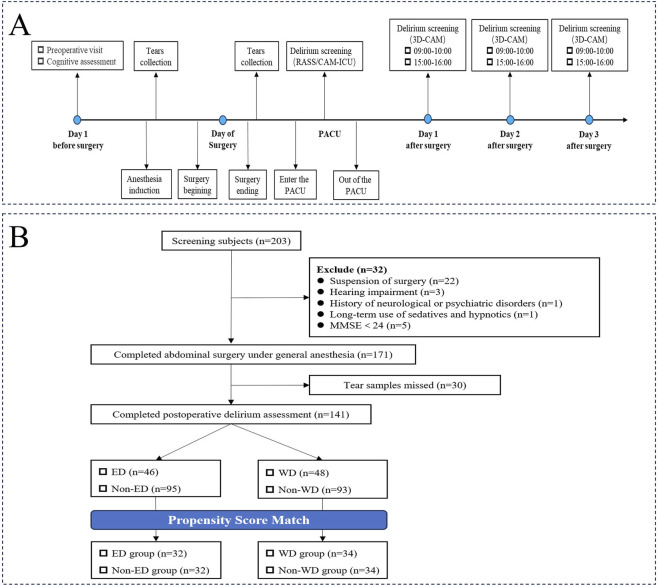
Study flow chart of baseline data collection, tear sample collection, and delirium assessment. **(A)** In this study, we collected patient tear samples twice during the anesthesia process and defined the collected samples after anesthesia induction as preoperative tears (post-induction tears), and the collected samples before the end of the surgical suture as postoperative tears (pre-emergence tears). For the postoperative delirium evaluation, we used the CAM-ICU scale combined with the RASS to evaluate ED occurring in the recovery room and 3D-CAM scale to assess WD 3 days after surgery. **(B)** Patient enrollment and matching flow chart. MMSE, Mini-Mental State Examination; ED, delirium after surgery during emergence; WD, delirium after surgery in ward.

This study’s primary endpoint was the incidence of postoperative delirium, which was evaluated by two trained anesthesiologists in both the post-anesthesia care unit (PACU) and general wards following surgery. Delirium assessment in the PACU employed the Richmond Agitation-Sedation Scale (RASS) combined with the CAM-ICU. The CAM-ICU was administered for delirium assessment only when the RASS score was ≥-3 (Card et al., 2015). Delirium assessment in the postoperative ward employed the 3D-CAM (Marcantonio et al., 2014). Figure 1A shows that delirium was assessed at 15 and 30 min after the patient was transferred to the PACU, at 9:00–10:00 and 15:00–16:00 daily in the ward for 3 days following surgery.

The diagnostic criteria for delirium comprise four key features: (Oh et al., 2017): acute onset with fluctuating course, (Zhang et al., 2020), inattention, (Neufeld et al., 2013), disorganized thinking, and (Sharma et al., 2005) altered level of consciousness. A definitive diagnosis of delirium is established when both Features 1 and 2 are present concurrently, accompanied by either Feature 3 or 4 (Mattison, 2020).

Standardized Management Strategies for Postoperative Delirium After Onset: Corresponding non-pharmacological interventions are implemented based on delirium subtypes. For hypoactive delirium, nursing interventions can be delivered by increasing sensory stimulation, including continuous interaction with family members and caregivers, as well as encouragement of early mobilization. In contrast, for hyperactive delirium, stimulus reduction strategies are recommended, such as avoiding confrontational approaches, playing soothing music, and dimming ambient light (Peden et al., 2021; Prandeh Afshar et al., 2025). For the ED analysis, the non-ED group comprised patients without ED, irrespective of whether WD subsequently occurred. For the WD analysis, the non-WD group comprised patients without WD, irrespective of whether ED had occurred previously. These analyses were designed to examine two temporally distinct delirium endpoints rather than mutually exclusive delirium subtypes.

### 2.4 Tear collection

All patients were collected 2 tear samples, the first tear sample was collected after anesthesia induction (post-induction sample), and the second tear sample was collected before completion of surgical suturing (late intraoperative/pre-emergence sample). These two time points were selected to provide a standardized perioperative baseline and an immediately pre-emergence perioperative state while minimizing variability related to postoperative interventions and sampling delay. Schirmer strips (SURGI EDGE, Ahmedabad, India) were used for tear sample collection. First, the lower eyelid of the patient was gently pulled open, and the Schirmer strip was carefully inserted into the conjunctival sac, ensuring no contact with the cornea or facial skin. After 5 min, the strip was removed and placed into an Eppendorf (EP) tube, which was then stored in the refrigerator at −80 °C until further processing.

### 2.5 Tear sample preparation and UHPLC-Orbitrap Exploris-MS analysis

The initial extraction was performed by adding 1,000 μL of methanol: acetonitrile: water (2:2:1, v/v) to each sample. After 30 s of vortex homogenization, the mixtures underwent 20 min of ice-cooled ultrasonication and 1 h stabilization at −40 °C. Phase separation was achieved by centrifugation at 12,000 rpm (13,800 × g, 4 °C, 15 min), with 800 μL supernatant collected for vacuum drying. The residues were then resolubilized in 100 μL of the same solvent system supplemented with isotopic internal standards, followed by 10 min ice-bath sonication post-vortexing. After final centrifugation under identical rotor conditions (12,000 rpm, 13,800 × g), 80 μL aliquots were prepared for LC-MS injection.

LC-MS/mass spectrometer (MS) analyses were conducted using a Vanquish ultra-high performance liquid chromatograph (Thermo Fisher Scientific). The target compounds were chromatography-separated through a UPLC BEH Amide liquid column (2.1 mm × 100 mm, 1.7 μm) coupled with an Orbitrap Exploris 120 mass spectrometer (Orbitrap MS, Thermo). The liquid chromatography phase A comprised an aqueous solution containing 25 mmol/L ammonia hydroxide and 25 mmol/L ammonium acetate, whereas phase B consisted of acetonitrile. The injection volume was set at 2 μL, and the auto-sampler temperature was maintained at 4 °C.

The Orbitrap Exploris 120 mass spectrometer, controlled by Xcalibur software (v4.4, Thermo), supported both MS1 and MS2 data acquisition. Key parameters included: sheath gas flow rate (50 Arb), auxiliary gas flow rate (15 Arb), capillary temperature (320 °C), full MS resolution (60,000), and MS/MS resolution (15,000). Collision energy was set at 10/30/60 (NCE mode), with spray voltages of 3.8 kV (positive) and −3.4 kV (negative).

### 2.6 The metabolite annotation procedure

After the assay was completed, the raw off-machine data were converted to mzXML format using ProteoWizard (Version 3.0.21229). The R package XCMS (Version 3.2) was then employed for data processing, including spectral deconvolution, peak detection, peak alignment, peak matching, quantification, and preliminary quality control. A data matrix containing retention time (RT), mass-to-charge ratio (m/z), and peak abundance was generated accordingly. Based on the XCMS outputs, the R package CAMERA (Version 3.16) was utilized for feature peak annotation and metabolite cluster merging analysis.

The peak data obtained above were subjected to preprocessing and filtration, with detailed steps as follows.

- Deviation filtration: Peaks with a relative standard deviation (RSD) > 30% in quality control (QC) samples were filtered out.
- Missing value filtration: Peaks with missing values (intensity = 0) in >50% of the samples were excluded.
- Missing value imputation: Missing values were imputed with half of the minimum intensity value.
- Normalization: Internal standard normalization was performed, and the resulting matrix was used for polar metabolomics analysis.

### 2.7 Statistical analysis and data processing

A total of 21,816 peaks were initially detected, and 20,064 metabolites were retained after preprocessing and quality control as described above. The processed dataset (containing sample names, peak numbers, and normalized areas) was analyzed using SIMCA16.0.2 (Sartorius Stedim Data Analytics AB). Data were log-transformed and scaled prior to principal component analysis (PCA), where samples beyond the 95% confidence interval in score plots were flagged as outliers.

### 2.8 Multivariate modeling and validation

Supervised orthogonal partial least squares-discriminant analysis (OPLS-DA) modeling was performed to discriminate sample groups and identify significant metabolites (VIP >1, p < 0.05 by t-test). The Benjamini–Hochberg method was also employed to correct p-values for multiple comparisons. Model robustness was verified through 7-fold cross-validation (R^2^Y and Q^2^ metrics) and permutation testing. The variable importance projection (VIP) values from the first principal component were used to evaluate metabolite contributions.

### 2.9 Pathway analysis

Biologically relevant pathways were interrogated using the Kyoto Encyclopedia of Genes and Genomes (KEGG) and MetaboAnalyst platforms, focusing on metabolites meeting both statistical significance (VIP >1, p < 0.05) and biological relevance criteria.

## 3 Statistical analysis

Data analysis was performed using SPSS 26.0 software, with a p value <0.05 considered statistically significantly different. Continuous variables were summarized as mean ± SD for normally distributed data or median (IQR) for non-normal distributions, while categorical variables were reported as frequencies (percentages). Normality was assessed using the Kolmogorov-Smirnov test. In the clinical analysis the primary aim was to select candidate patients for metabolomics through the baseline data. The propensity score matching was performed to balance baseline characteristics between groups with and without emergence delirium (ED) and WD, respectively. Baseline-matched factors included age, sex, body mass index, education level, medical history, ASA status, Huaxi Emotional-distress Index, MMSE score, use of anticholinergic drugs (yes/no), anesthesia duration, surgery duration, and use of PCIA (yes/no). The matching procedure employed a 1:1 nearest neighbor approach without replacement, utilizing a logit model with a caliper width of 0.05. Baseline characteristic differences between the matched groups were subsequently compared with those observed in the original unmatched cohort. In the un-matched cohort normal distributed data was compared using independent t-test, and abnormal distributed data was compared using Mann–Whitney U test. The categorical data was compared using chi-square test or Fisher’s exact test. And in matched cohort same statistical methods were performed. Additionally, the Boruta algorithm was utilized for parameter screening to develop an optimized model for predicting the presence or absence of ED and WD, and the receiver operating characteristic curve (ROC) and area under the curve (AUC) were calculated.

## 4 Results

Figure 1B shows that, in total, 203 patients were screened before surgery. Of these, 32 were excluded from the study, and tear samples were not collected for 30 during surgery. A total of 141 patients were finally included in the postoperative delirium assessment of this study, 66 patients (46.81%) did not develop delirium in any form following surgery, whereas 75 patients (53.19%) experienced at least one subtype of postoperative delirium, yielding an overall delirium incidence of 53.19%. Further stratification of the delirium cases showed that 46 patients had ED, with an incidence of 32.62%, and 48 patients had WD, with an incidence of 34.04%. A breakdown of the comorbidity patterns between the two subtypes revealed that 27 patients (58.70% of ED cases) had isolated ED without WD; 29 patients (60.42% of WD cases) had isolated WD without ED; and 19 patients had sequential onset of ED followed by WD, accounting for 41.30% of the ED cohort and 39.58% of the WD cohort, respectively (Supplementary Table S4). Accordingly, the ED and WD analyses should be interpreted as two related endpoint-specific analyses rather than as statistically independent comparisons. Metabolites identified in both analyses may reflect shared perioperative delirium vulnerability, whereas apparent differences between ED- and WD-associated signals should be interpreted cautiously. These findings are consistent with the ED incidence range of 37%–45% (Zhang et al., 2020; Neufeld et al., 2013; Sharma et al., 2005) and WD incidence range of 30.9%–36% (Sharma et al., 2005; Choi et al., 2017; Miyagawa et al., 2017; Kim et al., 2022) documented in prior clinical studies. Tables 1 and 2 present the demographic and baseline characteristics of these groups before and after matching.

**TABLE 1:** Demographic and clinical characteristics at baseline.

Variables	Unmatched cohort			Matched cohort		
	ED (n = 46)	Non-ED (n = 95)	Statistics	ED (n = 32)	Non-ED (n = 32)	Statistics
Sex Male Female	30 (62.5%) 16 (34.8%)	55 (57.9%) 40 (42.1%)	*X^2^* = 0.694 *P* = 0.405	20 (62.5%) 12 (37.5%)	21 (65.6%) 11 (34.4%)	*X^2^* = 0068 *P* = 0.794
Education Primary or below Secondary College or above	28 (60.9%) 13 (28.3%) 5 (10.9%)	41 (43.2%) 40 (42.1%) 14 (14.7%)	*X^2^* = 3.911 *P* = 0.141	16 (50.0%) 11 (34.4%) 5 (15.6%)	17 (53.1%) 12 (37.5%) 3 (9.4%)	*X^2^* = 0.574 *P* = 0.751
Medical history Yes No	37 (80.4%) 9 (19.6%)	69 (72.6%) 26 (27.4%)	*X^2^* = 1.011 *P* = 0.315	24 (75.0%) 8 (25.0%)	21 (65.6%) 11 (34.4%)	*X^2^* = 0.674 *P* = 0.412
ASA I II III	4 (8.7%) 26 (56.5%) 16 (34.8%)	15 (15.8%) 57 (60.0%) 23 (24.2%)	*X^2^* = 1.680 *P* = 0.290	3 (9.4%) 21 (65.6%) 8 (25.0%)	3 (9.4%) 20 (62.5%) 9 (28.1%)	*X^2^* = 0.083 *P* = 0.959
Anticholinergic agent Yes No	15 (32.6%) 31 (67.4%)	29 (30.5%) 66 (69.5%)	*X^2^* = 0.063 *P* = 0.802	9 (28.1%) 23 (71.9%)	10 (31.3%) 22 (68.8%)	*X^2^* = 0.075 *P* = 0.784
PCIA Yes No	43 (93.5%) 3 (6.5%)	83 (87.4%) 12 (12.6%)	*X^2^* = 1.217 *P* = 0.386	29 (90.6%) 3 (9.4%)	30 (93.8%) 2 (6.3%)	*X^2^* = 0.217 *P* = 0.641
Postoperative complication Yes No	1 (2.2%) 45 (97.8%)	5 (5.3%) 90 (94.7%)	*X^2^* = 0.726 *P* = 0.664	1 (3.1%) 31 (96.9%)	2 (6.3%) 30 (93.8%)	*X^2^* = 0.350 *P* = 0.554
Age (year)	72.00 ± 5.44	71.57 ± 4.79	*T* = 0.480 *P* = 0.632	72.22 ± 5.71	73.41 ± 5.80	*T* = 0.826 *P* = 0.412
Height(m)	1.62 ± 0.07	1.61 ± 0.08	*T* = 0.779 *P* = 0.438	1.63 ± 0.08	1.62 ± 0.08	*T* = 0.379 *P* = 0.706
Weight (kg)	60.33 ± 8.23	59.33 ± 10.25	*T* = 0.557 *P* = 0.565	61.56 ± 9.09	58.09 ± 9.96	*T* = 1.455 *P* = 0.151
BMI	23.05 ± 2.52	22.92 ± 3.31	*T* = 0.232 *P* = 0.817	23.28 ± 2.78	22.09 ± 2.75	*T* = 1.724 *P* = 0.090
HEI	0.0 (0.0–0.0)	1.0 (0.0–2.0)	*U = 1.237* *P* = 0.255	0.0 (0.0–1.0)	0.0 (0.0–1.0)	*U = 0.522* *P* = 0.602
MMSE	27.78 ± 2.15	27.86 ± 2.21	*T* = 0.205 *P* = 0.838	27.94 ± 2.27	27.41 ± 2.54	*T* = 0.882 *P* = 0.381
Duration of anesthesia (min)	272 (218–390)	225 (170–315)	*U = 2.592* *P* = 0.009	228 (181–384)	270 (203–333)	*U = 0.913* *P* = 0.361
Duration of surgery (min)	230 (175–322)	185 (130–270)	*U = 2.582* *P* = 0.012	188 (134–284)	228 (164–288)	*U = 1.296* *P* = 0.195

ED: delirium after surgery during emergence; WD: delirium after surgery in ward; ASA = American Society of Anesthesiologists; PCIA = patient-controlled intravenous Analgesia; BMI = body Mass Index; HEI = Huaxi Emotional-distress Index; MMSE = MiniMental state examination.

**TABLE 2:** Demographic and clinical characteristics at baseline.

Variables	Unmatched cohort			Matched cohort		
	WD (n = 48)	Non-WD (n = 93)	Statistics	WD (n = 34)	Non-WD (n = 34)	Statistics
Sex Male Female	31 (64.6%) 17 (35.4%)	54 (58.1%) 39 (41.9%)	*X^2^* = 0.562 *P* = 0.453	20 (58.8%) 14 (41.2%)	23 (67.6%) 11 (32.4%)	*X^2^* = 0.569 *P* = 0.451
Education Primary or below Secondary College or above	30 (62.5%) 14 (29.2%) 4 (8.3%)	39 (41.9%) 39 (41.9%) 15 (16.1%)	*X^2^* = 5.537 *P* = 0.063	20 (58.8%) 11 (32.4%) 3 (8.8%)	19 (55.9%) 9 (26.5%) 6 (17.6%)	*X^2^* = 1.226 *P* = 0.542
Medical history Yes No	37 (77.1%) 11 (22.9%)	69 (74.2%) 24 (25.8%)	*X^2^* = 0.142 *P* = 0.707	23 (67.6%) 11 (32.4%)	26 (76.5%) 8 (23.5%)	*X^2^* = 0.657 *P* = 0.417
ASA I II III	7 (14.6%) 25 (52.1%) 16 (33.3%)	12 (12.9%) 58 (62.4%) 23 (24.7%	*X^2^* = 2.192 *P* = 0.334	6 (17.6%) 17 (50.0%) 11 (32.4%)	4 (11.8%) 21 (61.8%) 9 (26.5%)	*X^2^* = 1.021 *P* = 0.600
Anticholinergic agent Yes No	12 (25.0%) 36 (75.0%)	32 (34.4%) 61 (65.6%)	*X^2^* = 1.305 *P* = 0.253	12 (35.3%) 22 (64.7%)	12 (35.3%) 22 (64.7%)	*X^2^* = 0.000 *P* = 1.000
PCIA Yes No	46 (95.8%) 2 (4.2%)	80 (86.0%) 13 (14.0%)	*X^2^* = 3.206 *P* = 0.073	32 (94.1%) 2 (5.9%)	30 (88.2%) 4 (11.8%)	*X^2^* = 0.731 *P* = 0.393
Postoperative complication Yes No	4 (8.3%) 44 (91.7%)	2 (2.2%) 91 (97.8%)	*X^2^* = 2.970 *P* = 0.085	2 (5.9%) 32 (94.1%)	0 (0.0%) 34 (100%)	*X^2^* = 0.515 *P* = 0.473
Age (year)	72.46 ± 5.49	71.32 ± 4.71	*T* = 0.122 *P* = 0.202	72.68 ± 5.04	72.53 ± 5.02	*T* = 0.121 *P* = 0.904
Height (m)	1.60 ± 0.07	1.62 ± 0.08	*T* = 1.672 *P* = 0.097	1.59 ± 0.07	1.63 ± 0.08	*T* = 2.112 *P* = 0.039
Weight (kg)	57.56 ± 10.47	60.73 ± 9.03	*T* = 1.869 *P* = 0.064	57.21 ± 11.92	59.76 ± 9.54	*T* = 977 *P* = 0.332
BMI	22.54 ± 3.46	23.18 ± 2.84	*T* = 1.166 *P* = 0.246	22.60 ± 3.70	22.55 ± 3.00	*T* = 0.067 *P* = 0.947
HEI	0.0 (0.0–2.0)	0.0 (0.0–2.0)	*U = 0.141* *P* = 0.887	1.0 (0.0–2.0)	0.0 (0.0–2.0)	*U = 0.237* *P* = 0.812
MMSE	26.73 ± 2.77	28.41 ± 1.53	*T* = 0.205 *P* = 0.838	27.53 ± 1.76	27.88 ± 1.74	*T* = 0.832 *P* = 0.409
Duration of anesthesia (min)	300 (187–394)	235 (182–290)	*U = 2.498* *P* = 0.012	265 (199–395)	289 (175–386)	*U = 0.288* *P* = 0.773
Duration of surgery (min)	260 (149–318)	190 (143–250)	*U = 2.121* *P* = 0.034	215 (165–318)	260 (128–306)	*U = 0.386* *P* = 0.699

Data are presented as mean ± standard deviation, number (percentage). ED: delirium after surgery during emergence; WD: delirium after surgery in ward; ASA, american society of anesthesiologists physical status; PCIA, Patient-Controlled Intravenous Analgesia; BMI, body mass index; HEI, Huaxi Emotional-distress Index; MMSE, MiniMental State Examination.

In this study, differential metabolites were selected based on VIP scores >1 and p-values <0.05. After propensity score matching, comparisons were conducted between the emergence delirium (ED) group and non-ED group. A total of 1,856 differential metabolites were identified in preoperative tear samples, whereas 826 differential metabolites were detected in postoperative tear samples. Parallel comparisons between the postoperative delirium (WD) group and non-WD group revealed 889 differential metabolites in preoperative tear samples and 724 differential metabolites in postoperative tear samples.

**FIGURE 2:**
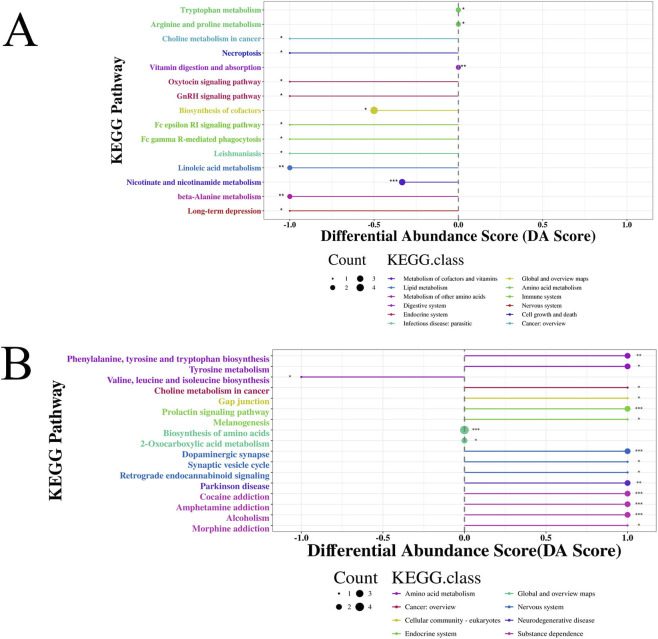
Differential metabolites and metabolic pathways associated with ED. **(A)** Metabolic pathways associated with ED in preoperative tears (Supplementary Table S3). **(B)** Metabolic pathways associated with ED in postoperative tears. “*” representing significant p value, * = 0.05, ** = 0.01, *** = 0.001; ED, delirium after surgery during emergence.

Analysis of the KEGG database revealed 18 significantly enriched metabolic pathways in the tears of the ED group before surgery (p < 0.05) (Figure 2A) (Supplementary Table S3). Among them, the decreased maleic and quinolinic acid levels, along with the increased niacinamide levels, were associated with alterations in the nicotinate and nicotinamide metabolic pathway. Additionally, decreased quinolinic acid levels and increased 2-aminobenzoic acid levels contributed to the disturbance of the tryptophan metabolic pathway. Decreased arachidonic acid (ARA) and 9-OxoODE levels led to the downregulation of the linoleic metabolic pathway (Supplementary Figure S1A) (Supplementary Table S1).

Seventeen metabolic pathways were significantly enriched in the tears of the ED group after surgery (Figure 2B) (Supplementary Table S3). A decrease in 2-isopropylmalic acid levels in the ED group was associated with the downregulation of the valine, leucine, and isoleucine biosynthesis pathway. Additionally, increased 2-aminobenzoic acid and L-tyrosine levels were associated with the upregulation of the phenylalanine, tyrosine, and tryptophan biosynthesis pathway Furthermore, elevated L-tyrosine and dopamine levels contributed to the upregulation of the tyrosine metabolic pathway (Supplementary Figure S1B) (Supplementary Table S1).

**FIGURE 3:**
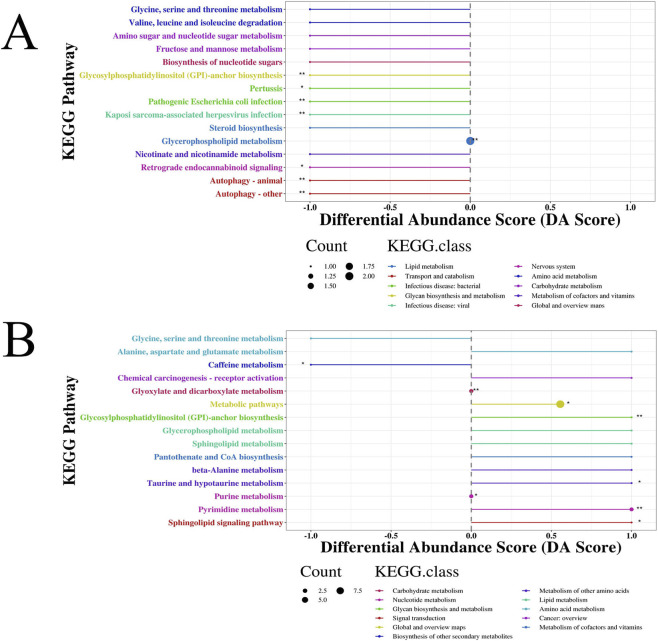
Differential metabolites and metabolic pathways associated with WD. **(A)** Metabolic pathways associated with WD in preoperative tears. **(B)** Metabolic pathways associated with WD in postoperative tears. “*” representing significant p value, * = 0.05, ** = 0.01, *** = 0.001; WD, Delirium after surgery in ward.

Eight pathways were significantly enriched in the tears of the WD group before surgery (Figure 3A) (Supplementary Table S3). The increased diethanolamine level and decreased glycerophospholipid metabolite PE (20:2 (11Z,14Z)/14:0) level in the WD group were associated with disturbances in the glycerophospholipid metabolic pathway (Supplementary Figure S2A) (Supplementary Table S1).

Eight metabolic pathways were significantly enriched in the tears of the WD group after surgery (Figure 3B) (Supplementary Table S3). Among them, increased uridine and uracil levels in the WD group were associated with the upregulation of the pyrimidine metabolism pathway. Conversely, decreased glyoxylic acid levels and increased oxalic acid levels were associated with the disturbance in the glyoxylate and dicarboxylate metabolic pathway (Supplementary Figure S2B) (Supplementary Table S1).

**FIGURE 4:**
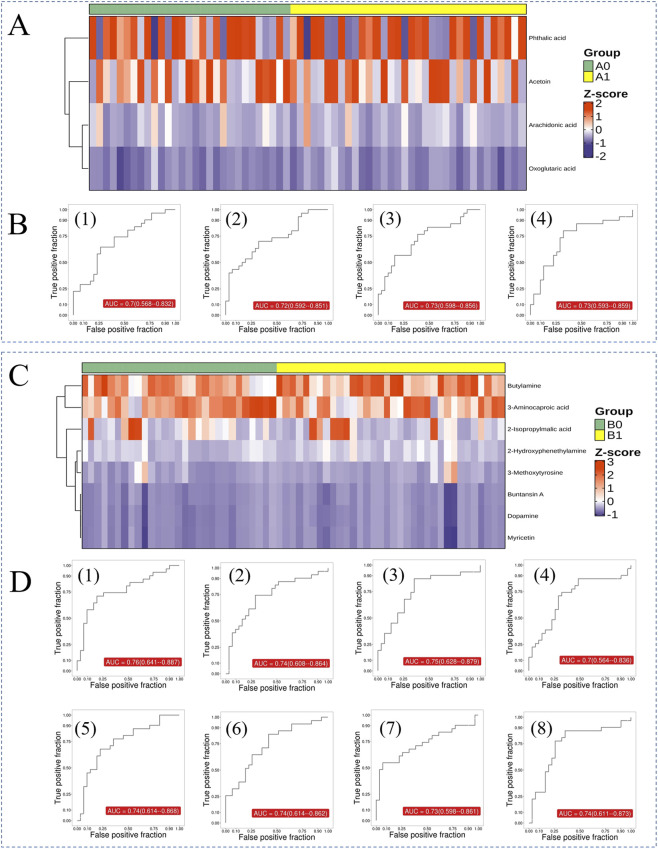
Differential metabolites that are predictive (AUC ≥0.7) of ED and their ROC. **(A)** Hierarchical clustering analysis heatmap of differential metabolite levels in preoperative tears, A0 = preoperative tears of non-ED group, A1 = preoperative tears of ED group. **(B)** 1. Phthalic acid; 2. Oxoglutaric acid; 3. Acetoin; 4. Arachidonic acid. **(C)** Hierarchical clustering analysis heatmap of differential metabolite levels in postoperative tears, B0 = postoperative tears of non-ED group, B1 = postoperative tears of ED group. **(D)** 1. Buntansin A; 2. Myricetin; 3. 3-Aminocaproic acid; 4. Dopamine; 5. 2-Hydroxyphenethylamine; 6. 3-Methoxytyrosine; 7. Butylamine; 8. 2-Hydroxyphenethylamine. ED, delirium after surgery during emergence; ROC, receiver operating characteristic curves; AUC, the area under the curve.

**FIGURE 5:**
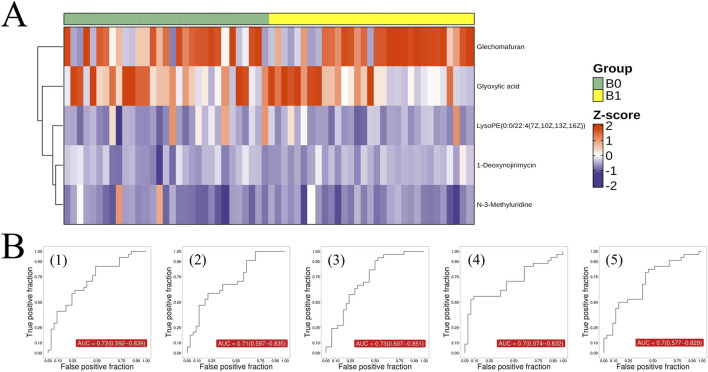
Differential metabolites that are predictive (AUC ≥0.7) of WD and their ROC. **(A)** Hierarchical clustering analysis of heatmap differential metabolite levels in postoperative tears, B0 = postoperative tears of non-WD group, B1 = postoperative tears of WD group. **(B)** 1. Glechomafuran; 2. N-3-Methyluridine; 3. Glyoxylic acid; 4. LysoPE (00,224 (7Z,10Z,13Z,16Z)); 5. 1-Deoxynojirimycin. WD, Delirium after surgery in ward; ROC, receiver operating characteristic curves; AUC, area under the curve.

To compare the predictive power of individual metabolites for delirium, we plotted ROC curves for each well-defined differential metabolite and calculated AUC (AUC >0.7). We ultimately identified 17 metabolites whose level changes were predictive of delirium (Supplementary Table S2). Among them, a decrease in ARA levels in tears before surgery (Figures 4A,B-4) and an increase in dopamine levels after surgery (Figures 4C,D-4) showed predictive efficacy for the occurrence of ED. However, a decrease in glyoxylic acid level after surgery (Figures 5A,B-3) demonstrated predictive value for WD.

**FIGURE 6:**
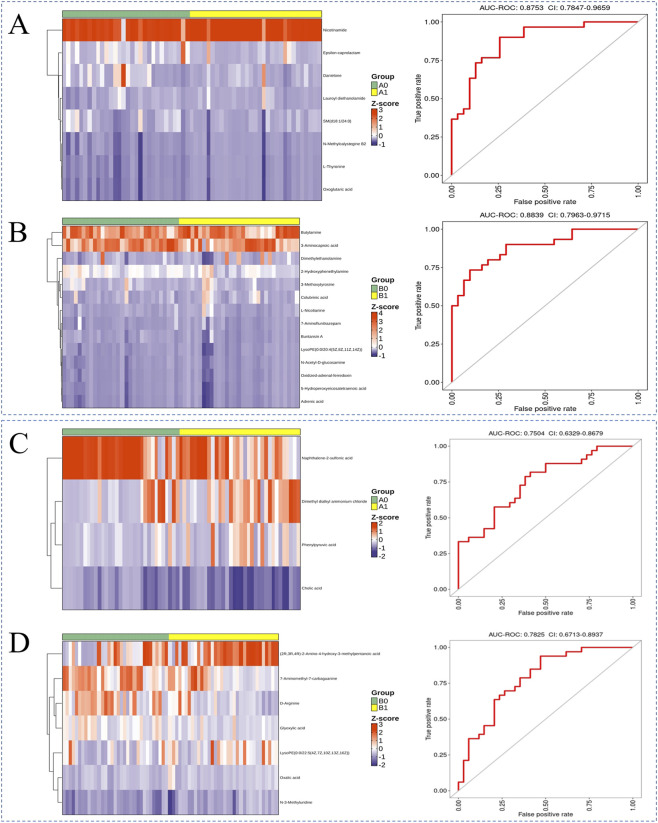
The characteristic metabolites contained in each subgroup were screened using the Boruta algorithm, while the comprehensive prediction model was established using these differential metabolites. **(A)** Preoperative tears comparison between ED and non-ED groups; **(B)** Postoperative tears comparison between ED and non-ED groups; **(C)** Preoperative tears comparison between WD and non-WD groups; **(D)** Postoperative tears comparison between WD and non-WD groups. ED, delirium after surgery during emergence; WD, delirium after surgery in ward; A0 = preoperative tears of non-delirium group, A1 = preoperative tears of the delirium group; B0 = postoperative tears of non-delirium group, B1 = postoperative tears of the delirium group; AUC-ROC, the area under the receiver operating characteristic curve.

Finally, to enhance the predictive efficiency of the model, we employed the Boruta algorithm to identify the characteristic metabolites from each subgroup and used them to construct a comprehensive delirium prediction model. All four delirium prediction models showed predictive efficacy for delirium occurrence. Among them, the comprehensive prediction model based on tear samples collected before (AUC = 0.88, 95% CI: 0.78–0.97) and after (AUC = 0.88, 95% CI: 0.80–0.97) surgery in the ED group demonstrated a high predictive value for ED occurrence. The predictive efficiency of the WD models reached 0.75 (95% CI: 0.63–0.87) and 0.78 (95% CI: 0.67–0.89), respectively (Figure 6). Because the matched datasets were relatively small (ED: 32 vs. 32; WD: 34 vs. 34), the Boruta algorithm was used to reduce the high-dimensional metabolite space before model construction. The exact numbers of Boruta-retained metabolites in the four models were 8, 14, 4, and 7, respectively, as detailed in Supplementary Table S5. Accordingly, these models should be interpreted as exploratory models derived from a limited matched cohort.

## 5 Discussion

In this study, alterations in linoleic acid (LA), branched-chain amino acids (BCAAs), aromatic amino acids (AAAs), tryptophan, and tyrosine metabolic pathways were associated with ED, whereas disturbances in glycerophospholipid pathway and glyoxylate and dicarboxylate pathway were linked to WD. At the metabolite level, decreased ARA and increased dopamine were observed in ED, while reduced glyoxylic acid was associated with WD. These findings suggest that tear metabolite profiles may provide insight into perioperative metabolic alterations related to delirium vulnerability.

The essential omega-6 fatty acid LA is a crucial component of human biochemistry. Although LA is generally considered a precursor to ARA, most LA in the brain is not converted into ARA but is oxidized and decomposed through fatty acid β-oxidation (DeMar et al., 2006). LA contributes to the regulation of key cellular stress responses: oxidative stress, endoplasmic reticulum stress, and inflammation (Kim and Song, 2023). Studies show that docosapentaenoic acid, a metabolite of LA, can reduce the inflammatory response, thereby improving the neurodegeneration of mice in advanced Alzheimer’s disease models (Ma et al., 2020). Alarcon et al. revealed that LA might have a neuroprotective and anti-inflammatory effect in cells and Parkinson’s disease mouse models through its antioxidant properties, stimulation of lipid droplet (LD) formation, and induction of lipophagy (Alarcon-Gil et al., 2022). Additionally, a large plasma metabolomics study revealed a negative association between LA and sphingomyelin levels with dementia risk (Qiang et al., 2024). In our study, we found that the downregulation of LA metabolism before surgery is related to ED development.

Collectively known as BCAAs, valine, leucine, and isoleucine account for nearly 30% of the body’s amino acid pool. Beyond their role as protein building blocks, these essential amino acids regulate key metabolic processes through signaling functions, and can serve as an important energy source for the body during fasting (Le Couteur et al., 2020). Additionally, BCAAs are associated with aging and age-related syndromes, and studies show that their deficiency may significantly increase the risk of progression from mild cognitive impairment to AD in patients (Le Couteur et al., 2020; Ikeuchi et al., 2022). AAAs, including tyrosine, phenylalanine, and tryptophan, are precursors for important neurotransmitters such as catecholamines and serotonin in the brain. BCAAs and AAAs are classified as large neutral amino acids (LNAAs) that compete for transport across the blood-brain barrier via shared LNAA transporters. An altered plasma BCAAs/AAAs ratio may lead to a neurotransmitter disorder in the brain, contributing to the pathological mechanisms underlying delirium (Wilson et al., 2020; Fernstrom, 2013). In the Successful Aging after Elective Surgery study, Tripp et al. employed plasma-targeted metabolomics technology to determine that the biosynthesis pathway of valine, leucine, and isoleucine was downregulated in patients with postoperative delirium (Tripp et al., 2021). Similarly, Guo et al. used serum metabolomics technology to show that leucine levels increased before but not after surgery, whereas phenylalanine and tyrosine levels increased before or after surgery in elderly patients with delirium following hemiarthroplasty (Guo et al., 2019). In this study, we observed downregulation of the postoperative biosynthesis for valine, leucine, and isoleucine, alongside upregulation of phenylalanine, tyrosine, and tryptophan biosynthesis in the ED group. Additionally, the preoperative tryptophan metabolic pathway was disturbed in the ED group, while the postoperative tyrosine metabolic pathway was upregulated in the ED group. This further suggests that abnormal metabolism of BCAAs and AAAs, along with neurotransmitter disturbances, contribute to delirium occurrence.

ARA is an Ω-6 polyunsaturated fatty acid with a dual role in neurobiology. As an important component of the membrane phospholipids, ARA supports nerve growth and synaptic regeneration. Conversely, ARA serves as a precursor of eicosanoic acids, including prostaglandins, thromboxane A2, and leukotrienes, which play a double-edged role (primarily proinflammation) in the neuroinflammation process. As a result, the comprehensive effect of ARA in the pathological processes of neurodegenerative diseases remains controversial (Regulska et al., 2021; Kursu et al., 2022). Tan et al. observed in a hair metabolomics study that ARA metabolism was disturbed in an Aβ_1-42_-induced rat model of AD, with ARA levels significantly reduced in the AD model rats than in the sham-fed rats (Tan et al., 2023). Ishikura et al. and Tokuda et al. found that dietary supplementation with ARA may improve cognitive ability in elderly individuals (Ishikura et al., 2009; Tokuda et al., 2014). In our study, we observed that ARA concentration in tears before surgery decreased in the ED group. The delirium prediction model incorporating ARA suggests that the decrease in ARA levels before surgery in elderly patients may be associated with delirium occurrence. Therefore, dietary supplementation with ARA may be a feasible strategy for preventing postoperative delirium.

It is widely accepted that peripheral dopamine cannot penetrate the blood-brain barrier under normal conditions. However, when cerebral vascular permeability is altered, and the blood-brain barrier is damaged, dopamine may enter the brain (Sommer et al., 2002). Excessive dopamine levels are considered important pathological mechanisms of delirium (Maldonado, 2013; Yilmaz et al., 2016). However, clinical studies investigating the prevention and treatment of delirium using antipsychotic drugs, primarily function by blocking dopamine D2-type receptors, have not strongly supported this theory (Agar et al., 2017; van den Boogaard et al., 2018). In our study, we observed that the dopamine concentration in postoperative tears was significantly increased in the ED group. Moreover, the delirium prediction model incorporating dopamine levels demonstrated a certain predictive efficacy for ED, further supporting the association between increased dopamine concentrations and the incidence of delirium.

Neuroinflammation is an important pathological mechanism that underlies delirium development. Glycerophospholipid is an essential neuronal membrane constituent, and its homeostasis is critical for maintaining nerve cell membrane fluidity, integrity, and overall function. Additionally, they serve as a precursor for lipid mediators (Frisardi et al., 2011). Several animal studies show that disruptions in glycerophospholipid metabolism are involved in the pathological processes of depression and AD (Qian et al., 2023; Zhao et al., 2021; Zheng et al., 2021; Xie et al., 2023). Han et al. conducted metabolomics and lipidomics analysis on preoperative CSF samples from elderly patients with hip fractures, revealing that neuroinflammation resulting from disturbances in glycerophospholipid and sphingolipid metabolism is associated with postoperative delirium (Han et al., 2020). In our study, we observed that a disorder in glycerophospholipid metabolism in tears before surgery was associated with WD occurrence.

Glyoxylic acid is an intermediate product in the glyoxylic acid cycle, which allows certain organisms to convert fatty acids into carbohydrates, and the conjugated base of glyoxylic acid is glyoxylate. In a plasma metabolomics study on exposure-induced panic attacks, Martins et al. show that glyoxylate exhibits significant dynamic changes during an anxiety attack, with baseline levels correlating with the severity of anxiety experienced by patients during exposure (Martins et al., 2019). Glyoxylate and dicarboxylate metabolism is involved in carbohydrate metabolism and are linked to the tricarboxylic acid cycle through oxaloacetic acid. Studies show that disturbances in glyoxylate and dicarboxylate metabolic pathways are significant in metabolomic studies of AD and diabetic cognitive dysfunction (Yu et al., 2017; Zhao et al., 2022). Han et al. conducted a multi-omics study on neurocognitive disorders (NCDs) in the elderly, revealing that disturbances in glyoxylate and dicarboxylate metabolism may serve as risk predictors of NCDs (Han et al., 2022). In our study, we observed that a decrease in glyoxylic acid levels and disturbance of glyoxylate and dicarboxylate metabolic pathway in postoperative tears in the WD group is associated with delirium occurrence.

Studies have established some prediction models on blood and CSF metabolomics, as well as EEG of WD. The AUC of ROC for the delirium prediction model established in the blood metabolomics studies of Guo et al. and Tripp et al. are 0.77 and 0.838, respectively (Guo et al., 2019; Tripp et al., 2021). Additionally, Pan et al. developed a prediction model and an enhanced version in CSF metabolomics analysis, achieving AUC values of 0.75 and 0.8 (Pan et al., 2019), whereas Han et al. created several prediction models in CSF metabolomics and lipidomics analysis, achieving a maximum AUC value of 0.92 (Han et al., 2020). Koch et al. conducted an EEG study on WD and found that a preoperative spectral edge frequency cut-off value of 17.75HZ yields an ROC AUC of 0.718 (Koch et al., 2021). Pollak et al. established an EEG prediction model for WD with an AUC of 0.73 (Pollak et al., 2024). In this study, we developed a comprehensive prediction model for WD. The preoperative and postoperative tear prediction models achieved AUCs of 0.75 and 0.78, respectively. This is comparable to the prediction model from blood and CSF metabolomics studies and superior to the EEG model. Additionally, to our knowledge, this study is the first to employ metabolomics to investigate ED. The comprehensive tear metabolomics prediction model developed in this study demonstrates excellent predictive efficacy for ED, with AUC of 0.88 and 0.88 before and after surgery, respectively.

Adequate pain control is crucial after abdominal surgery and may strongly influence delirium. In the present study, TAP block - a nerve block technique, is often used in combination with general anesthesia to reduce the stress response and perioperative opioid use (Liu et al., 2019; Liu et al., 2022)- was implemented as the primary multimodal analgesic strategy, with PCIA available as an optional supplementary approach (Sammons and Ritchey, 2015). Although laparoscopic surgery, widely used in colon cancer radical resection for its small incisions and mild trauma, is less invasive, postoperative delirium still occurs in approximately 50% of patients undergoing laparoscopic abdominal surgery (Lin et al., 2021).

The second tear sample was collected before completion of surgical suturing and therefore represented a late intraoperative/pre-emergence sample rather than a true postoperative sample. This distinction is important for interpretation. For ED, which was assessed shortly after PACU arrival, the second sampling point may reflect biology more proximal to symptom onset. In contrast, for WD, which was assessed during the postoperative ward period, this sampling point should be interpreted more cautiously as reflecting perioperative vulnerability or early pathophysiological change rather than the direct metabolic state of established ward delirium.

The present study should be interpreted primarily as exploratory. Although the identified tear-metabolite pathways may reflect biologically relevant processes involved in perioperative delirium, the specific metabolite signatures and the observed model performance were derived from a single-center cohort of elderly patients undergoing abdominal surgery under a relatively standardized perioperative pathway. Their applicability to other surgical populations, institutions, and perioperative management settings remains unknown.

## 6 Limitations

As with all studies, certain limitations should be noted. First, while we successfully identified metabolic pathway changes associated with postoperative delirium in tears and developed predictive models, the small sample size raises the potential for Type I and II errors. Because ED and WD were not mutually exclusive and some patients contributed to both endpoint-specific analyses, the identified metabolomic signatures may include both shared delirium-vulnerability signals and phase-specific features. This overlap limits direct inference regarding strict subtype specificity. Additionally, the prediction model lacks external validation, which limits our conclusions and the portability and generalizability of our findings. Notably, this study prioritized surgical factors while not specifically accounting for the impact of patients’ comorbidities, certain comorbidities may exert an impact on basal metabolic status. In the present study, we detected tear metabolomic profiles both preoperatively and postoperatively, with a particular focus on the perioperative changes in tear metabolites as potential predictive factors for postoperative delirium. Although perioperative changes in tear metabolites may be clinically informative, baseline diagnostic heterogeneity and comorbidity-related metabolic differences may also influence the observed metabolomic profiles and should be addressed in future studies. Primary diagnosis may have influenced baseline and perioperative tear-metabolomic profiles through differences in inflammatory activity, tumor burden, nutritional status, and procedure-related physiology. Because diagnosis was not explicitly modelled in the present analysis, residual diagnosis-related confounding cannot be excluded. Therefore, the identified tear-metabolite signals should be interpreted as delirium-associated findings within this abdominal-surgery cohort rather than diagnosis-independent biomarkers. Further investigations are warranted in future studies to explore the influence of comorbidities on metabolic profiles among elderly patients undergoing abdominal surgery. Second,a notable limitation of the present study is the relatively high incidence of postoperative delirium among participants, which may be attributable to the single-center design and short 5–6 months recruitment period—factors that could restrict the generalizability of our findings due to limited sample diversity and potential selection bias. Consequently, the proposed prediction model requires further validation through large-sample, multicenter studies to enhance its external validity. Third, our study exclusively included people undergoing abdominal surgery. Therefore, further verification is required for other surgical types. Furthermore, despite the use of feature selection, cross-validation, and permutation testing, the possibility of model overfitting cannot be excluded because the matched subgroup sizes were small relative to the number of candidate metabolites. Therefore, the predictive performance reported here should be considered preliminary and requires validation in independent external cohorts.

## 7 Conclusion

In this study, tear metabolomics identified candidate metabolites and metabolic pathways associated with emergence delirium and ward delirium in elderly patients undergoing abdominal surgery. These findings support the feasibility of tears as a noninvasive source for perioperative biomarker discovery; however, the predictive models remain preliminary and require multicenter external validation before clinical application.

## Data Availability

The data presented in the study are deposited in the scienceDB repository, under the DOI: 10.57760/sciencedb.37534.
