# Supplementary material for: Predictive role of tear metabolomics in delirium during anesthesia emergence and postoperative period in elderly patients after abdominal surgery
Source: Front Mol Biosci. 2026 Jun 4;13:1705024. doi: 10.3389/fmolb.2026.1705024 (PMC13275471; doi:10.3389/fmolb.2026.1705024)
Supplement: Supplementary file 1 [file Table1.docx]

| Supplementary Table 1：Important pathways and their metabolites | | | | | | |  |
| --- | --- | --- | --- | --- | --- | --- | --- |
|  |  |  |  |  |  |  |  |
| **Group** | **pathway** | **metabolites** | **VIP** | **p-value** | **fc** | **L-fc** |  |
| ED (A) : Non-ED (A) | Nicotinate and nicotinamide metabolism | Maleic acid | 1.32 | 0.024 | 0.89 | -0.16 |  |
|  |  | Niacinamide | 1.09 | 0.009 | 2.44 | 1.29 |  |
|  |  | Quinolinic acid | 1.18 | 0.034 | 0.68 | -0.56 |  |
|  | Tryptophan metabolism | 2-Aminobenzoic acid | 1.49 | 0.002 | 3.16 | 1.66 |  |
|  |  | Quinolinic acid | 1.18 | 0.034 | 0.68 | -0.56 |  |
|  | Linoleic acid metabolism | Arachidonic acid | 1.44 | 0.004 | 0.66 | -0.60 |  |
|  |  | 9-OxoODE | 1.76 | 0.031 | 0.73 | -0.46 |  |
| ED (B) : Non-ED (B) | Valine, leucine and isoleucine biosynthesis | 2-Isopropylmalic acid | 1.11 | 0.010 | 0.63 | -0.67 |  |
|  | Phenylalanine, tyrosine and tryptophan biosynthesis | 2-Aminobenzoic acid | 1.95 | 0.013 | 2.41 | 1.27 |  |
|  |  | L-Tyrosine | 1.25 | 0.037 | 2.46 | 1.30 |  |
|  | Tyrosine metabolism | L-Tyrosine | 1.25 | 0.037 | 2.46 | 1.30 |  |
|  |  | Dopamine | 2.00 | 0.006 | 1.71 | 0.77 |  |
| PD (A) : Non-PD (A) | Glycerophospholipid metabolism | Diethanolamine | 2.10 | 0.049 | 1.22 | 0.28 |  |
|  |  | PE(20:2(11Z,14Z)/14:0) | 1.19 | 0.046 | 0.57 | -0.82 |  |
| PD (B) : Non-PD (B) | Pyrimidine metabolism | Uridine | 1.67 | 0.042 | 1.42 | 0.50 |  |
|  |  | Uracil | 1.65 | 0.019 | 2.13 | 1.09 |  |
|  | Glyoxylate and dicarboxylate metabolism | Glyoxylic acid | 1.24 | 0.039 | 0.90 | -0.15 |  |
|  |  | Oxalic acid | 1.97 | 0.012 | 1.26 | 0.33 |  |
| ED = emergence delirium; PD = delirium in the postoperative ward; (A) = Preoperative tear ; (B) = Postoperative tear; VIP = Variable Importance in the Projection; fc = Fold Change; L-fc = Flod Change takes the logarithm base 2 | | | | | | |  |
|  |  |  |  |  |  |  |  |
