# Supplementary material for: Predictive role of tear metabolomics in delirium during anesthesia emergence and postoperative period in elderly patients after abdominal surgery
Source: Front Mol Biosci. 2026 Jun 4;13:1705024. doi: 10.3389/fmolb.2026.1705024 (PMC13275471; doi:10.3389/fmolb.2026.1705024)
Supplement: Supplementary file 2 [file Table2.docx]

| Supplementary Table 2: Differential metabolites with potential predictive value for delirium | | | | | | | |  |
| --- | --- | --- | --- | --- | --- | --- | --- | --- |
| **Group** | **metabolites** | **AUC** | **ci(0.95)** | **VIP** | **p-value** | **fc** | **L-fc** |  |
| ED (A) : Non-ED (A) | Acetoin | 0.73 | 0.598--0.856 | 1.93 | 0.001 | 0.49 | -1.02 |  |
|  | Arachidonic acid | 0.73 | 0.593--0.859 | 1.44 | 0.004 | 0.66 | -0.60 |  |
|  | Oxoglutaric acid | 0.72 | 0.592--0.851 | 2.03 | 0.011 | 0.54 | -0.88 |  |
|  | Phthalic acid | 0.70 | 0.568--0.832 | 2.10 | 0.005 | 1.85 | 0.89 |  |
| ED (B) : Non-ED (B) | 2-Isopropylmalic acid | 0.74 | 0.611--0.873 | 1.11 | 0.010 | 0.63 | -0.67 |  |
|  | Buntansin A | 0.76 | 0.641--0.887 | 1.22 | 0.000 | 0.47 | -1.09 |  |
|  | Myricetin | 0.74 | 0.608--0.864 | 1.02 | 0.011 | 0.61 | -0.72 |  |
|  | 2-Hydroxyphenethylamine | 0.74 | 0.614--0.868 | 1.35 | 0.007 | 1.55 | 0.63 |  |
|  | 3-Aminocaproic acid | 0.75 | 0.628--0.879 | 1.46 | 0.001 | 1.83 | 0.88 |  |
|  | 3-Methoxytyrosine | 0.74 | 0.614--0.862 | 1.96 | 0.002 | 1.73 | 0.79 |  |
|  | Butylamine | 0.73 | 0.598--0.861 | 2.08 | 0.001 | 2.95 | 1.56 |  |
|  | Dopamine | 0.70 | 0.564--0.836 | 2.00 | 0.006 | 1.71 | 0.77 |  |
| WD (B) : Non-WD (B) | Glyoxylic acid | 0.73 | 0.607--0.851 | 1.24 | 0.039 | 0.90 | -0.15 |  |
|  | 1-Deoxynojirimycin | 0.70 | 0.577--0.829 | 2.75 | 0.003 | 1.87 | 0.90 |  |
|  | Glechomafuran | 0.72 | 0.592--0.839 | 2.84 | 0.009 | 2.34 | 1.22 |  |
|  | LysoPE(0:0/22:4(7Z,10Z,13Z,16Z)) | 0.70 | 0.574--0.832 | 1.91 | 0.001 | 2.24 | 1.16 |  |
|  | N-3-Methyluridine | 0.71 | 0.587--0.835 | 1.66 | 0.016 | 1.83 | 0.87 |  |
| ED = emergence delirium; WD: delirium after surgery in ward ; (A) = Preoperative tear ; (B) = Postoperative tear; AUC = Area under the receiver operating characteristic curve; ci(0.95) = 95% confidence interval; VIP = Variable Importance in the Projection; fc = Fold Change; L-fc = Flod Change takes the logarithm base 2 | | | | | | | |  |
|  |  |  |  |  |  |  |  |  |
|  |  |  |  |  |  |  |  |  |
