# Supplementary material for: Predictive role of tear metabolomics in delirium during anesthesia emergence and postoperative period in elderly patients after abdominal surgery
Source: Front Mol Biosci. 2026 Jun 4;13:1705024. doi: 10.3389/fmolb.2026.1705024 (PMC13275471; doi:10.3389/fmolb.2026.1705024)
Supplement: Supplementary file 3 [file Table5.docx]

| **Supplementary Table 5: Metabolites selected for modeling by the Boruta algorithm** | | | | | | | |
| --- | --- | --- | --- | --- | --- | --- | --- |
| **Group** | **MS2.name** | **fc** | **l-fc** | **p-value** | **VIP** | **Imp** | **BS** |
| ED (A) : Non-ED (A) | Nicotinamide | 1.82 | 0.86 | 0.033 | 1.73 | 7.88 | 1 |
|  | L-Thyronine | 0.33 | -1.59 | 0.012 | 1.72 | 3.13 | 1 |
|  | Oxoglutaric acid | 0.54 | -0.88 | 0.011 | 2.08 | 4.62 | 1 |
|  | SM(d18:1/24:0) | 0.58 | -0.78 | 0.079 | 0.92 | 2.79 | 1 |
|  | Epsilon-caprolactam | 0.76 | -0.39 | 0.515 | 1.74 | 4.03 | 1 |
|  | Lauroyl diethanolamide | 0.70 | -0.51 | 0.022 | 1.87 | 4.11 | 1 |
|  | N-Methylcalystegine B2 | 0.72 | -0.48 | 0.408 | 1.69 | 4.29 | 1 |
|  | Danielone | 0.42 | -1.25 | 0.032 | 1.55 | 4.40 | 1 |
| ED (B) : Non-ED (B) | Dimethylethanolamine | 3.08 | 1.62 | 0.089 | 1.03 | 2.80 | 1 |
|  | 3-Methoxytyrosine | 1.74 | 0.80 | 0.002 | 2.61 | 3.54 | 1 |
|  | Butylamine | 2.91 | 1.54 | 0.001 | 1.35 | 7.74 | 1 |
|  | 5-Hydroperoxyeicosatetraenoic acid | 2.05 | 1.04 | 0.039 | 0.54 | 3.27 | 1 |
|  | 3-Aminocaproic acid | 1.81 | 0.86 | 0.001 | 0.89 | 6.73 | 1 |
|  | 2-Hydroxyphenethylamine | 1.53 | 0.61 | 0.010 | 1.07 | 2.69 | 1 |
|  | Oxidized-adrenal-ferredoxin | 1.91 | 0.93 | 0.004 | 1.83 | 2.78 | 1 |
|  | LysoPE(0:0/20:4(5Z,8Z,11Z,14Z)) | 1.71 | 0.77 | 0.038 | 0.82 | 3.59 | 1 |
|  | Buntansin A | 0.47 | -1.09 | 0.000 | 1.19 | 6.64 | 1 |
|  | Adrenic acid | 0.62 | -0.70 | 0.004 | 1.89 | 3.40 | 1 |
|  | L-Nicotianine | 1.85 | 0.89 | 0.049 | 2.51 | 3.67 | 1 |
|  | 7-Aminoflunitrazepam | 1.00 | 0.00 | 0.995 | 1.79 | 3.72 | 1 |
|  | N-Acetyl-D-glucosamine | 1.78 | 0.83 | 0.016 | 1.53 | 5.25 | 1 |
|  | Colubrinic acid | 2.71 | 1.44 | 0.014 | 0.41 | 3.93 | 1 |
| WD (A) : Non-WD (A) | Naphthalene-2-sulfonic acid | 0.54 | -0.88 | 0.108 | 2.42 | 3.51 | 1 |
|  | Phenylpyruvic acid | 1.59 | 0.67 | 0.007 | 2.14 | 7.38 | 1 |
|  | Dimethyl dialkyl ammonium chloride | 1.28 | 0.36 | 0.248 | 2.52 | 2.28 | 1 |
|  | Cholic acid | 0.46 | -1.12 | 0.011 | 0.55 | 4.20 | 1 |
| WD (B) : Non-WD (B) | (2R,3R,4R)-2-Amino-4-hydroxy-3-methylpentanoic acid | 2.60 | 1.38 | 0.071 | 2.79 | 5.02 | 1 |
|  | Glyoxylic acid | 0.90 | -0.15 | 0.040 | 1.38 | 8.21 | 1 |
|  | 7-Aminomethyl-7-carbaguanine | 0.93 | -0.10 | 0.670 | 1.58 | 3.06 | 1 |
|  | LysoPE(0:0/22:5(4Z,7Z,10Z,13Z,16Z)) | 1.79 | 0.84 | 0.036 | 2.56 | 3.77 | 1 |
|  | Oxalic acid | 1.26 | 0.33 | 0.012 | 1.70 | 2.62 | 1 |
|  | N-3-Methyluridine | 1.83 | 0.87 | 0.016 | 1.30 | 6.14 | 1 |
|  | D-Arginine | 0.56 | -0.82 | 0.012 | 2.59 | 2.09 | 1 |
| ED: delirium after surgery during emergence；WD: delirium after surgery in the ward; (A): Preoperative tear; (B): Postoperative tear; fc: Fold Change; L-fc: Flod Change takes the logarithm base 2; VIP: Variable Importance in the Projection; lmp: The feature importance assigned by the Boruta algorithm, NA indicates that the metabolite was filtered out during variance analysis and collinearity analysis. BS: The final decision of the Boruta algorithm, 1 indicates that the metabolite is important and can be used for subsequent modeling. | | | | | | | |
