# Supplementary material for: Predictive role of tear metabolomics in delirium during anesthesia emergence and postoperative period in elderly patients after abdominal surgery
Source: Front Mol Biosci. 2026 Jun 4;13:1705024. doi: 10.3389/fmolb.2026.1705024 (PMC13275471; doi:10.3389/fmolb.2026.1705024)
Supplement: Supplementary file 4 [file Table3.docx]

| Supplementary Table 3：Detailed information on significantly enriched metabolic pathways (p＜0.05) | | | | | | | |  |
| --- | --- | --- | --- | --- | --- | --- | --- | --- |
|  |  |  |  |  |  |  |  |  |
| **Group** | **Pathway** | **cpd.(all)** | **cpd.(dem)** | **p_value** | **up_nums** | **down_nums** | **DA_score** |  |
| ED (A) : Non-ED (A) | Nicotinate and nicotinamide metabolism | 8 | 3 | 7.92E-04 | 1 | 2 | -0.33 |  |
|  | Linoleic acid metabolism | 8 | 2 | 4.02E-03 | 0 | 2 | -1 |  |
|  | beta-Alanine metabolism | 7 | 2 | 5.23E-03 | 0 | 2 | -1 |  |
|  | Vitamin digestion and absorption | 5 | 2 | 7.71E-03 | 1 | 1 | 0 |  |
|  | GnRH signaling pathway | 2 | 1 | 2.06E-02 | 0 | 1 | -1 |  |
|  | Leishmaniasis | 1 | 1 | 2.06E-02 | 0 | 1 | -1 |  |
|  | Biosynthesis of cofactors | 18 | 4 | 2.26E-02 | 1 | 3 | -0.5 |  |
|  | Arginine and proline metabolism | 13 | 2 | 2.49E-02 | 1 | 1 | 0 |  |
|  | Fc gamma R-mediated phagocytosis | 2 | 1 | 2.74E-02 | 0 | 1 | -1 |  |
|  | Long-term depression | 1 | 1 | 3.08E-02 | 0 | 1 | -1 |  |
|  | Tryptophan metabolism | 4 | 2 | 3.24E-02 | 1 | 1 | 0 |  |
|  | Necroptosis | 2 | 1 | 3.41E-02 | 0 | 1 | -1 |  |
|  | Fc epsilon RI signaling pathway | 2 | 1 | 3.75E-02 | 0 | 1 | -1 |  |
|  | Choline metabolism in cancer | 6 | 1 | 3.75E-02 | 0 | 1 | -1 |  |
|  | Oxytocin signaling pathway | 1 | 1 | 4.08E-02 | 0 | 1 | -1 |  |
|  | Amoebiasis | 2 | 1 | 4.42E-02 | 0 | 1 | -1 |  |
|  | Platelet activation | 1 | 1 | 4.75E-02 | 0 | 1 | -1 |  |
|  | Regulation of lipolysis in adipocytes | 3 | 1 | 4.75E-02 | 0 | 1 | -1 |  |
| ED (B) : Non-ED (B) | Cocaine addiction | 2 | 2 | 8.02E-05 | 2 | 0 | 1 |  |
|  | Biosynthesis of amino acids | 20 | 4 | 8.18E-05 | 2 | 2 | 0 |  |
|  | Amphetamine addiction | 2 | 2 | 1.37E-04 | 2 | 0 | 1 |  |
|  | Alcoholism | 3 | 2 | 1.71E-04 | 2 | 0 | 1 |  |
|  | Prolactin signaling pathway | 2 | 2 | 2.09E-04 | 2 | 0 | 1 |  |
|  | Dopaminergic synapse | 2 | 2 | 2.51E-04 | 2 | 0 | 1 |  |
|  | Parkinson disease | 3 | 2 | 1.22E-03 | 2 | 0 | 1 |  |
|  | Phenylalanine, tyrosine and tryptophan biosynthesis | 3 | 2 | 2.08E-03 | 2 | 0 | 1 |  |
|  | Tyrosine metabolism | 8 | 2 | 1.06E-02 | 2 | 0 | 1 |  |
|  | Melanogenesis | 1 | 1 | 1.24E-02 | 1 | 0 | 1 |  |
|  | Morphine addiction | 2 | 1 | 1.65E-02 | 1 | 0 | 1 |  |
|  | Gap junction | 1 | 1 | 2.26E-02 | 1 | 0 | 1 |  |
|  | Choline metabolism in cancer | 6 | 1 | 2.26E-02 | 1 | 0 | 1 |  |
|  | Synaptic vesicle cycle | 2 | 1 | 2.47E-02 | 1 | 0 | 1 |  |
|  | 2-Oxocarboxylic acid metabolism | 12 | 2 | 2.97E-02 | 1 | 1 | 0 |  |
|  | Retrograde endocannabinoid signaling | 3 | 1 | 3.88E-02 | 1 | 0 | 1 |  |
|  | Valine, leucine and isoleucine biosynthesis | 7 | 1 | 4.68E-02 | 0 | 1 | -1 |  |
| WD (A) : Non-WD (A) | Glycerophospholipid metabolism | 11 | 2 | 3.31E-03 | 1 | 1 | 0 |  |
|  | Autophagy - other | 1 | 1 | 4.84E-03 | 0 | 1 | -1 |  |
|  | Pathogenic Escherichia coli infection | 1 | 1 | 4.84E-03 | 0 | 1 | -1 |  |
|  | Glycosylphosphatidylinositol (GPI)-anchor biosynthesis | 2 | 1 | 6.45E-03 | 0 | 1 | -1 |  |
|  | Kaposi sarcoma-associated herpesvirus infection | 1 | 1 | 8.06E-03 | 0 | 1 | -1 |  |
|  | Autophagy - animal | 1 | 1 | 9.66E-03 | 0 | 1 | -1 |  |
|  | Pertussis | 1 | 1 | 1.61E-02 | 0 | 1 | -1 |  |
|  | Retrograde endocannabinoid signaling | 3 | 1 | 3.03E-02 | 0 | 1 | -1 |  |
| WD (B) : Non-WD (B) | Glyoxylate and dicarboxylate metabolism | 6 | 2 | 6.80E-03 | 1 | 1 | 0 |  |
|  | Pyrimidine metabolism | 9 | 2 | 7.24E-03 | 2 | 0 | 1 |  |
|  | Glycosylphosphatidylinositol (GPI)-anchor biosynthesis | 2 | 1 | 8.29E-03 | 1 | 0 | 1 |  |
|  | Purine metabolism | 8 | 2 | 1.68E-02 | 1 | 1 | 0 |  |
|  | Sphingolipid signaling pathway | 4 | 1 | 3.08E-02 | 1 | 0 | 1 |  |
|  | Metabolic pathways | 155 | 9 | 3.30E-02 | 7 | 2 | 0.56 |  |
|  | Caffeine metabolism | 1 | 1 | 4.48E-02 | 0 | 1 | -1 |  |
|  | Taurine and hypotaurine metabolism | 4 | 1 | 4.68E-02 | 1 | 0 | 1 |  |
| ED, delirium after surgery during emergence; WD, Delirium after surgery in ward; (A) = Preoperative tear ; (B) = Postoperative tear; cpd.(all) = The number of all metabolites detected in the pathway; cpd.(dem) = The number of differentially expressed metabolites detected in the pathway; up_nums = The number of upregulated, differentially expressed metabolites; down_nums = The number of downregulated, differentially expressed metabolites; DA_score = Differential Abundance Score. | | | | | | | |  |
|  |  |  |  |  |  |  |  |  |
|  |  |  |  |  |  |  |  |  |
