# Supplementary material for: Predictive role of tear metabolomics in delirium during anesthesia emergence and postoperative period in elderly patients after abdominal surgery
Source: Front Mol Biosci. 2026 Jun 4;13:1705024. doi: 10.3389/fmolb.2026.1705024 (PMC13275471; doi:10.3389/fmolb.2026.1705024)
Supplement: Supplementary file 5 [file Table4.docx]

| **Supplementary** **Table 4. Distribution of Subtypes among Patients with Postoperative Delirium** | | | | | |
| --- | --- | --- | --- | --- | --- |
| **Proportion Among Patients Who Completed Delirium Assessment (n=141)** | **Patients Assessed for Delirium (n=141)** | | | | |
|  | **Delirium = 75**  **（53.19%）** | | | | **Non-Delirium = 66（46.81%）** |
|  | **ED^+^ = 46（32.62%）** | | **WD^+^ = 48（34.04%）** | |  |
|  | **ED^+^ and WD^-^=27（19.15%）** | **ED^+^ and WD^+^ = 19（13.48%）** | | **ED^-^ and WD^+^=29（20.57%）** |  |
| **Proportion Within Each Subgroup (ED^+^ group = 46, WD^+^ group = 48)** | **Within the ED^+^ group** | | **Within the WD^+^ group** | |  |
|  | **27/46**  **(58.70%)** | **19/46**  **(41.30%)** | **19/48**  **(39.58%)** | **29/48**  **(60.42%)** |  |
| **ED^+^ = delirium after surgery during emergence; ED^-^ = patients without delirium after surgery during emergence; WD^+^ = delirium after surgery in ward; WD^-^ = patients without delirium after surgery in ward** | | | | | |
